# A Rare Case of Hydrochlorothiazide-Induced Hemolytic Anemia

**DOI:** 10.7759/cureus.17453

**Published:** 2021-08-26

**Authors:** Pilar Stevens-Cohen, Fardad Zaghi, Lawrence Zhu

**Affiliations:** 1 Cardiology, Mount Sinai South Nassau, Oceanside, USA; 2 Internal Medicine, Coney Island Hospital, Brooklyn, USA; 3 Osteopathic Medicine, New York Institute of Technology, Old Westbury, USA

**Keywords:** immune hemolytic anemia, drug-induced immune hemolytic anemia, hydrochlorothiazide, adverse drug event, immune-mediated hemolysis

## Abstract

Drug-induced immune hemolytic anemia is an exceedingly rare adverse drug event. Thiazide diuretics, commonly used in the treatment of primary hypertension, have been associated with this complication. In this case report, we present a 77-year-old male who developed acute hemolytic anemia two days after starting hydrochlorothiazide in the treatment of high blood pressure.

## Introduction

Drug-induced hemolytic anemia is classified as an immune-mediated reaction. This type of hemolysis is mediated by the attachment of immune complexes to red blood cell (RBC) membranes. Although hydrochlorothiazide (HCTZ) induced hemolytic anemia is uncommon, a few cases of variable severity have been reported [1-3]. In this case report, we aim to present an acute drug-induced immune hemolytic anemia (DIIHA) after two days of HCTZ use.

## Case presentation

A 77-year-old white male with a past medical history of coronary artery disease (CAD), hypertension, atrial fibrillation (AFib), and hyperlipidemia, and a past surgical history of coronary artery bypass graft (CABG) seven months prior, presented to the emergency department (ED) with severe malaise, chills, shortness of breath, jaundice, and dark urine. The patient's home medication consisted of lisinopril (20 mg), rosuvastatin (40 mg), aspirin (81 mg), and clopidogrel (75 mg). Two days prior, the patient was seen by his cardiologist and was prescribed HCTZ 12.5 mg. He took HCTZ for two days before developing these symptoms. The patient self-discontinued the medication at the onset of symptoms. The patient was seen by his primary care doctor one week prior to his ED admissions, during which the patient was asymptomatic.

On admission, patient’s complete blood count showed a white blood cell (WBC) count of 5.95 x 10^9^/L (with 85.8% neutrophils), hemoglobin (Hgb) of 9.2 g/dL (normal range: 13.5-17.5 g/dL), hematocrit (Hct) of 27.4% (normal range: 41%-50%), reticulocyte count of 8.5% (normal range: 0.5%-2.5%) with a corrected reticulocyte count of 5.17%, platelet count of 134 x 10^3^/µL (normal range: 150-450 x 10^3^/µL), mean corpuscular volume (MCV) of 89.89 fL (normal range: 80-100 fL), and red cell distribution width (RDW) of 14.4% (normal range: 12.2%-16.1%). The patient had a blood urea nitrogen (BUN) of 44 mg/dL (normal range: 7-20 mg/dL) and creatinine (Cr) of 1.0 mg/dL (normal range: 0.7-1.2 mg/dL). His liver enzymes, aspartate aminotransferase (AST) and alanine aminotransferase (ALT), were 72 U/L (normal range: 5-40 U/L) and 20 U/L (normal range: 7-55 U/L), respectively. Total bilirubin was 14.4 mg/dL (normal range: 0-1.2 mg/dL); with the direct bilirubin at 2.10 mg/dL (normal range: 0.1-0.3 mg/dL) and indirect bilirubin at 12.3 mg/dL (normal range: 0.2-0.8 mg/dL). Alkaline phosphatase was 98 U/L (normal range: 20-140 U/L), lactate dehydrogenase (LDH) was 591 U/L (normal range: 140-280 U/L), haptoglobin was < 1 mg/dL (normal range: 40-200 mg/dL), iron was 197 mcg/dL (normal range: 60-170 mcg/dL), transferrin was 152 mg/dL (normal range: 204-360 mg/dL), transferrin saturation was 91% (normal range: 15%-50%), ferritin was 2256 ng/mL (normal range: 12-300 ng/mL), vitamin B12 was 491 pg/mL (normal range: 190-950 pg/mL), and folate 3.7 ng/mL (normal range: 2.7-17.0 ng/mL). However, according to his primary care, the patient had lab work done one week prior and all his results were unremarkable; his hemoglobin was 13.3 g/dL and total bilirubin was 0.7 mg/dL. In addition, a computed tomography (CT) scan of the abdomen was suggestive of a side-branch intraductal papillary mucinous neoplasm (IPMN) of the pancreas measuring < 10 mm, which was determined to have a low risk for malignancy and will require a repeat CT scan in one year. The patient’s Coombs test was negative. A peripheral blood smear was performed the following day, which showed bands and atypical lymphocytes, but otherwise normal WBC morphology, no nucleated RBCs, 0-2 schistocytes per high power field, normocytic RBCs, no bite cells, mild rouleaux formation, and no platelet clumping. Given the patient’s history, presentation, and lab results, he was admitted to inpatient medicine and a hematology-oncology consult was requested. Cardiology was also consulted given his history of AFib and CAD, and mildly elevated cardiac enzymes (troponin of 0.04 ng/mL).

**Figure 1:**
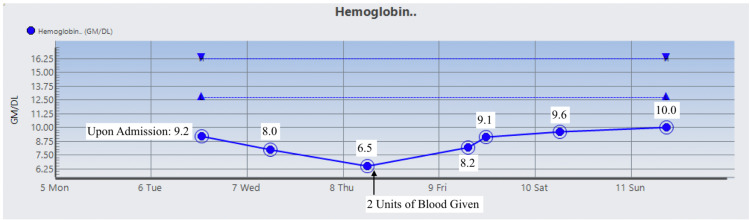
Patient's hemoglobin trend.

**Figure 2:**
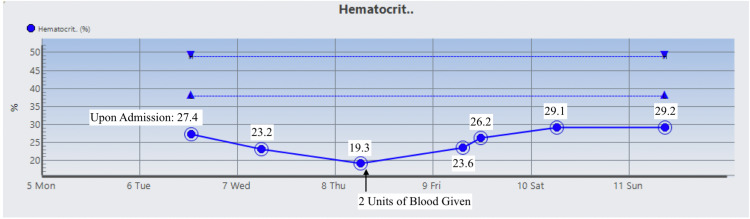
Patient's hematocrit trend.

**Figure 3:**
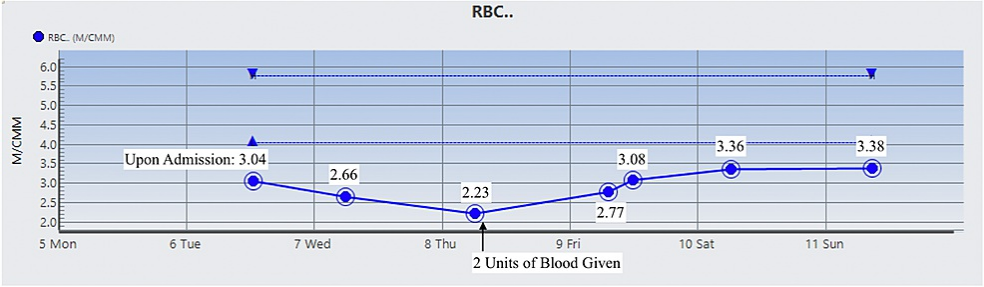
Patient's red blood cell (RBC) count trend.

**Figure 4:**
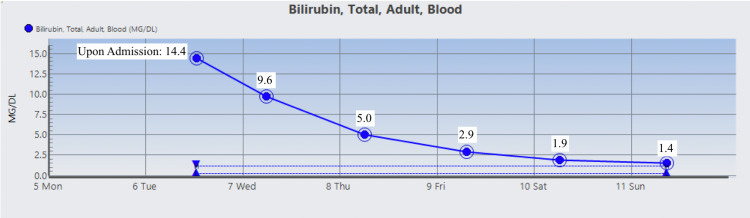
Patient's total bilirubin trend.

**Figure 5:**
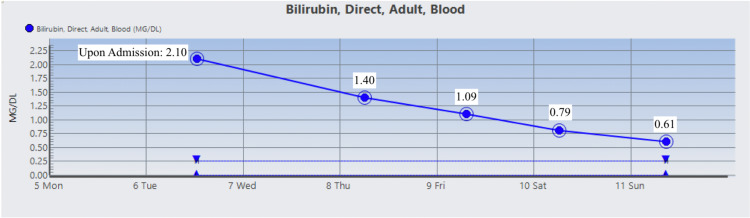
Patient's direct bilirubin trend.

**Figure 6:**
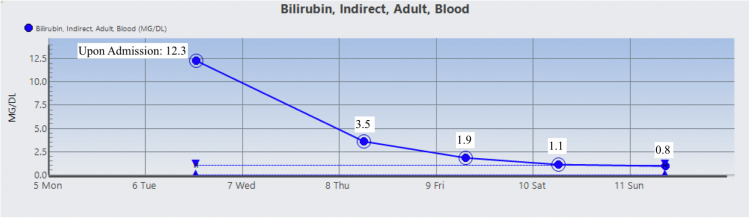
Patient's indirect bilirubin trend.

**Figure 7:**
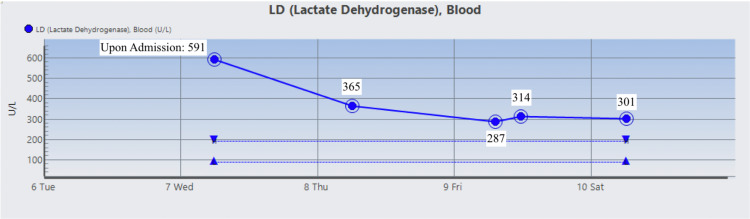
Patient's lactate dehydrogenase (LDH) trend.

The patient was started on Prednisone 1 mg/kg daily and two doses of intravenous immunoglobulin (IVIG). On day 3 , the patient’s Hgb dropped to 6.5 g/dL warranting two units of RBC suspension which raised his Hgb and Hct to 9.1 g/dL and 26.2%, respectively. His vitamin B12 level was normal, but folic acid was found to be low and ferritin level was high which prompted folic acid supplementation. During his stay, Hgb, Hct, RBC count, total bilirubin, direct bilirubin, indirect bilirubin, and LDH were monitored and their trends showed improvement as indicated in Figures 1-7. Upon discharge, the patient was doing well and was advised to avoid thiazide diuretics and sulfa drugs. His hypertension management now consisted of diet modification and metoprolol.

## Discussion

DIIHA has been estimated to occur in one in a million patients per year [4]. Although exceedingly rare, DIIHA can be potentially fatal even without properly establishing a definitive diagnosis. Drug-induced hemolytic anemia is exhibited in a variety of conditions such as G6PD deficiency in which vulnerable erythrocytes are subjected to oxidative damage, drug-induced thrombotic microangiopathy, and immune-mediated hemolytic anemia (IHA). The first documented case was in 1953 to mephenytoin (Mesantoin®) [5], a hydantoin that was used as an anticonvulsant. Since then, more than 120 different drugs have been associated with DIIHA [6,7]. The largest series of cases of DIIHA was reported in the Berlin Case-Control Surveillance Study in which the development of IHA was associated with the use of beta-lactam antibiotics [8].

There are two types of DIIHA: drug-dependent and drug-independent. The serological differentiation between the antibodies found in each type is essential in determining management. Drug-dependent antibodies (DDABs) yield a positive direct antiglobulin test (DAT) with negative elution, thus making the DAT a keystone test in the diagnostic work-up of DIIHA. Still, many DDABs can behave like an auto-antibody via the indirect antiglobulin test methods (eg, positive serum or eluate reactivity) when the drug or drug-antibody complexes are still present in the blood. In-depth drug history is essential to any case of hemolysis [9]. In DIIHA, intravascular hemolysis occurs as a result of complement activation via the binding of the offending agent to the RBC membrane. The lysis of RBCs elevates LDH and bilirubin levels and results in anemia. In severe cases, organ failure may be observed [10].

In our case, there was high suspicion for DIIHA, despite a negative DAT test, because the patient developed anemia, malaise, shortness of breath, dark urine, and jaundice two days after starting HCTZ with the absence of these symptoms nine days prior. The patient's lab results also exhibited a continued fall in Hgb values despite the administration of RBC suspensions. In addition, the patient's hyperbilirubinemia, hemoglobinuria (cause of the dark urine), low haptoglobin levels, high ferritin levels, and anemia findings suggested intravascular hemolysis. Even though a positive DAT, also known as a Coombs test, is a keystone in diagnosing these types of hemolysis, a negative Coombs test does not rule out the diagnosis [11]. The direct Coombs test in the immune complex type of drug-induced hemolytic anemia is usually positive with anti-C3 antisera, but negative with anti IgG antisera. The indirect Coombs test is positive only if the drug and complement are added to the reaction mixture; under those circumstances, the RBCs have C3 on the surface, but no detectable IgG. Antibodies may also be detectable due to their ability to immunologically hemolyze the RBCs; this type of hemolysis may be enhanced by utilizing the RBCs of individuals with paroxysmal nocturnal hemoglobinuria. For many years, it was believed that the drug did not react with the RBCs and that the red cells were “innocent bystanders.” Research now suggests that the reaction is a typical hapten reaction in which the drug forms a loose complex with membrane glycoproteins [12]. Even if the DAT is negative, additional diagnostic tests are indicated based on the patient’s history and physical exam findings, which is evident in our case.

Incidents of hemolytic anemia associated with the use of HCTZ have been documented in several instances. The first documented case was in 1976 in which Vila et al. [1] observed hemolytic episodes in a 67-year-old patient treated with a combination of methyldopa and HCTD for hypertension over a four-year period. They associated mild to moderate hemolytic anemia with the thiazide portion of the drug regimen. In 1980, Garratty et al. [2] documented a severe immune hemolytic anemia and acute renal failure in a 24-year old black patient who ingested 15-20 tablets of the same combination of drugs to commit suicide. They also determined that the hemolysis occurred via an immune-mediated mechanism against HCTZ. In both cases of intravascular hemolysis, antibodies were developed against HCTZ, but not against methyldopa. DIIHA, unlike other immune cytopenias, is poorly characterized and can go undiagnosed which can easily become fatal. Mortality in autoimmune hemolytic anemias is reported to be 11% [13,14]. The first recorded death associated with hemolytic anemia due to HCTZ use was reported in 1984 [3]. A 53-year-old black male expired 18 months after initiating methyldopa and HCTZ combination therapy. The patient had a positive direct and indirect Coombs test, a haptoglobin level at a low of 0.5 g/dL, and a high LDH. The actual cause of death was not established during the autopsy but was considered to be a fatal IHA associated with HCTZ use. It was not until 1986 that alternative diuretic therapies were offered to patients when Shirley et al. [15] characterized similar antibodies against diuretics such as HCTZ in serologic studies of a 53-year-old black female.

Even though healthcare is improving drastically day by day and diagnostic tests are becoming more sensitive and specific, the diagnosis of DIIHA may be delayed by 1-3 weeks after initiation of a drug in non-severe cases. The anemia may be misattributed to other causes without an established diagnosis [16]. In 2016, an 80-year-old male patient with moderate anemia, a mild increase in LDH, and a positive Coombs test was not diagnosed until 20 days after using a combination of angiotensin receptor blockers and HCTZ to treat hypertension [17]. Therefore, practitioners should have a high clinical suspicion for DIIHA in patients with a significant medical history and clinical signs and symptoms of anemia after starting medications that are associated with DIIHA irrespective of Coomb's test results. Cessation of the offending agent in mild cases and corticosteroids in addition to supportive therapy in more severe cases are recommended. Our patient recovered after stopping the HCTZ, and steroid, IVIG, and supportive therapy were initiated. The patient was asked to avoid thiazide diuretics and sulfa drugs indefinitely.

## Conclusions

DIIHA is a rare adverse drug event, but, because of its variable presentation, a negative Coombs test does not rule out the diagnosis. Healthcare providers should maintain a high clinical suspicion in patients with a significant medical history and clinical signs and symptoms of anemia after starting medications that are known to be associated with DIIHA. Treatment involves stopping the offending agent and initiating supportive care. Steroids and IVIG may have some efficacy in treatment on a case-by-case basis.
